# A home-based EEG neurofeedback treatment for chronic neuropathic pain—a pilot study

**DOI:** 10.3389/fpain.2025.1479914

**Published:** 2025-03-11

**Authors:** Mohamed Sakel, Christine A. Ozolins, Karen Saunders, Riya Biswas

**Affiliations:** ^1^ East Kent Neuro-Rehabilitation Service, East Kent Hospitals University NHS Foundation Trust, Canterbury, United Kingdom; ^2^ Exsurgo Ltd., Auckland, New Zealand; ^3^ Centre for Health Services Studies, University of Kent, Canterbury, United Kingdom

**Keywords:** chronic neuropathic pain, EEG neurofeedback, home-based neurofeedback, brain-computer interface, neuromodulation, neuropathic pain, remote telehealth

## Abstract

**Objective:**

This study assessed the effect of an 8-week home-based neurofeedback intervention in chronic neuropathic pain patients.

**Subjects/Patients:**

A cohort of eleven individuals with chronic neuropathic pain receiving treatment within the NHS framework.

**Methods:**

Participants were trained to operate a home-based neurofeedback system. Each received a portable Axon system for one week of electroencephalogram (EEG) baselines, followed by an 8-week neurofeedback intervention, and subsequent 12 weeks of follow-up EEG baselines. Primary outcome measures included changes in the Brief Pain Inventory and Visual Analogue Pain Scale at post-intervention, and follow-ups compared with the baseline. Secondary outcomes included changes in depression, anxiety, stress, pain catastrophizing, central sensitization, sleep quality, and quality of life. EEG activities were monitored throughout the trial.

**Results:**

Significant improvements were noted in pain scores, with all participants experiencing overall pain reduction. Clinically significant pain improvement (≥30%) was reported by 5 participants (56%). Mood scores showed a significant decrease in depression (*p* < 0.05), and pain catastrophizing (*p* < 0.05) scores improved significantly at post-intervention, with continued improvement at the first-month follow-up.

**Conclusion:**

The findings indicate that an 8-week home-based neurofeedback intervention improved pain and psychological well-being in this sample of chronic neuropathic pain patients. A randomized controlled trial is required to replicate these results in a larger cohort.

**Clinical Trial Registration:** https://clinicaltrials.gov/study/NCT05464199, identifier: (NCT05464199).

## 1 Introduction

Chronic pain (CP) is a disease defined as persistent or recurrent pain lasting for three months or more, resulting in substantial economic and healthcare burdens, and often leading to prolonged disability and substantial quality of life deficits (1, 2). Reviews suggest a considerable proportion of the UK population (approximately 28 million adults) live with CP conditions (3). Reduced mobility, sleep disturbance, anxiety, depression and stress commonly co-occur (4–7), producing significant impacts on psychological health and daily functioning (8, 9). The broad spectrum of symptom presentation and overlapping CP subtypes necessitate the development of effective management strategies that incorporate the biological, psychological, and social aspects of the disease.

While the precise economic burden of CP in the UK is not known, estimates reveal escalating costs for pharmacological treatments (10, 11), despite concerns around efficacy, and the dangers associated with prolonged use of analgesics, in particular opioids (12, 13). In 2020, the World Health Organization recognized CP as a distinct disease state (14), emphasizing the need for non-pharmacological person-centered treatment protocols to address the complex nature of the disease.

Chronic neuropathic pain (CNP) is a commonly occurring condition, affecting approximately 8% of the UK population (3). CNP is associated with damage to the somatosensory nervous system, causing burning, squeezing or pricking-type sensations, numbness, and allodynia, producing ongoing pain fluctuating in frequency and severity, and sensory hypersensitivity (15).

Electroencephalogram (EEG) studies have identified differential brain activity patterns in CP sufferers compared to healthy controls, particularly within the alpha (8–13 Hz), theta (4–8 Hz), and beta (13–30 Hz) frequency bands, characterized by suppressed alpha power and distinct rhythmic fluctuation in the theta and beta bands (16–20). Several comprehensive reviews of EEG studies have highlighted a body of evidence for suppressed alpha activity and increased beta and/or theta activity in CP patients (17, 18, 21). EEG neurofeedback (NFB) is a non-invasive neuromodulation technique that allows unconscious brain activity to become observable, enabling a person to consciously interact with brain activity involved in pain perception and regulation. NFB uses operant conditioning to facilitate Hebbian learning by rewarding sustained neural activity in frequencies associated with (in this case) relaxation and lowered pain states, which can strengthen existing neural pathways, create new ones, and encourage neuroplastic changes to brain structure and function (22).

EEG NFB protocols have been developed by researchers to successfully modulate brain activity involved in pain processing, with results suggesting NFB is a safe and effective treatment for the management of many CP conditions (21, 23–26). There have been a small number of studies using NFB to treat CNP that have produced symptom relief (21, 27, 28). For instance, one of the studies used a self-managed NFB intervention in CNP patients, which resulted in significant pain reduction via upregulation of the alpha frequency band at C4. Twelve out of fifteen participants achieved a statistically significant reduction in pain, eight of which also achieved a clinically significant reduction in pain (≥30%) (28). However, there is a need for larger and higher-quality trials within healthcare systems incorporating correlational analyses of changes in EEG with outcome measures to further evidence the efficacy of this intervention and provide objective measurements alongside subjective changes.

A recent proof-of-concept study evaluated the safety and efficacy of the Axon home-based EEG NFB system during the COVID-19 pandemic, showing significant improvements in pain, central sensitization, and quality of life measures (29). This current trial explored the concept of a home-based NFB intervention in a pilot cohort within the UK National Health Service (NHS) and expanded on the previous findings by measuring resting state EEG before, during, and after the NFB intervention. Feasibility and safety were also measured, the results of which have been published elsewhere (30).

The purpose of this study was to examine the efficacy of an 8-week home-based NFB intervention in individuals living with CNP within the NHS treatment framework. The primary hypotheses of this study were; (a) 8 weeks of usual care plus NFB would result in a reduction in pain (primary outcome) and improvements in secondary outcomes (mood, sleep, central sensitization, quality of life; (b) baseline EEG resting-state activity would change over the course of the intervention and; (c) changes in resting-state EEG would be associated with changes in the primary and secondary outcome measures.

## 2 Materials and methods

A prospective, open-label, single-arm, feasibility trial was approved by the London—Central Research Ethics Committee Health Research Authority Ethics (Reference: 20/LO/0523, IRAS reference number: 310674). The trial was registered with ClinicalTrials.gov (NCT05464199) and the Clinical Trials Unit of the sponsor organization: East Kent University Hospitals NHS Foundation Trust (EKHUFT) (Reference: 2022/CTU9/NEURO).

### 2.1 Setting and participants

**Figure 1:**
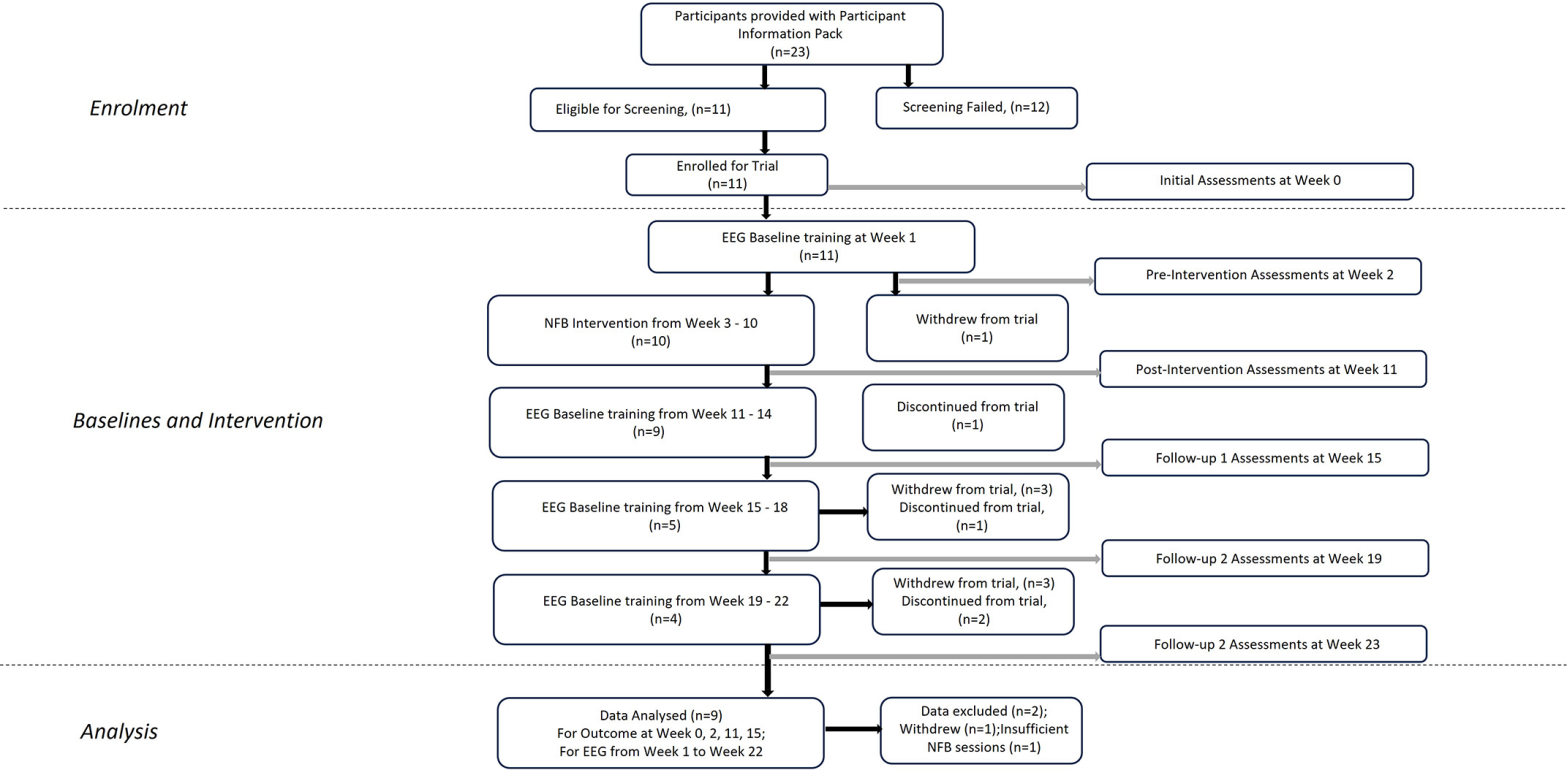
Participants flowchart throughout the trial.

**Figure 2:**
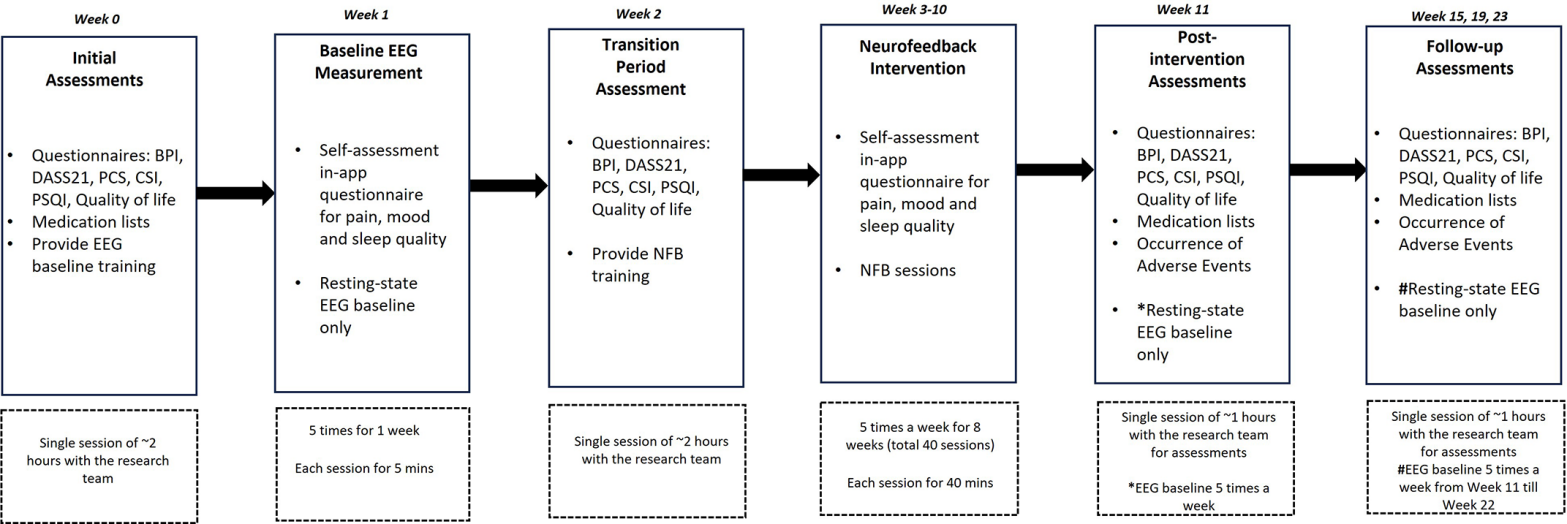
Study design and timelines. Study assessment sessions and training with the research team were conducted online via an internet-based video call. Feasibility and adverse events were assessed throughout the trial. Primary and secondary clinical outcome measurements were collected at Weeks 0, 2, 11, 15, 19 and 23. EEG data were collected from Week 1 to Week 22. BPI, brief pain inventory; DASS21, depression, stress and anxiety scale 21; PCS, pain catastrophizing scale; CSI, central sensitization inventory; PSQI, Pittsburgh sleep quality index; EEG, electroencephalography; NFB, neurofeedback.

Eleven participants (6 female) with a mean age of 51.89 (±6.85) years were recruited via NHS Outpatient clinics held by the Principal Investigator at EKHUFT and through social media (Figure 1). Prospective participants received comprehensive information, including a Participant Information Sheet, Consent Form, Headset Measurement Guide information sheet, trial flyer, and pre-screening information questionnaire. Upon confirming eligibility based on the pre-screening questionnaire against trial inclusion/exclusion criteria (Table 1), providing head circumference measurement, and expressing interest in trial enrolment, individuals proceeded to an initial assessment appointment conducted via secure video conference (Zoom). During this appointment, the process of gaining informed consent was undertaken, baseline outcome measurement questionnaires were completed, and headset measurements were obtained. The participants selected were considered to have intractable CNP and accompanying complex comorbidities and for whom previous interventions had been unsuccessful. All screening, consent, assessment, and training sessions with researchers were conducted online. A schematic representation of the study components is depicted in Figure 2. Pharmacological treatments were to be kept stable throughout the study period.

**Table 1:** Inclusion and exclusion criteria for this study.

Inclusion criteria	• 18 years and over • Ongoing chronic neuropathic pain for at least three months • Average pain rating in the last week of ≥4/10 • Head circumference of 520–620 mm • Access to reliable internet connection and Wi-Fi at home
Exclusion criteria	• Previous NFB training • Serious head injury (within 12 months) • Traumatic brain injury, concussion, major neurological disorder (e.g., trigeminal neuralgia), history of seizures, psychiatric disorder • Implanted electronic neuromodulation device • Implanted pacemaker or loop recorder • Inability to provide informed consent and any change in medication or treatment plan in the 1 week prior, during the intervention period, or in 12 weeks post-intervention, while EEG baselines are being recorded.

### 2.2 Equipment

**Figure 3:**
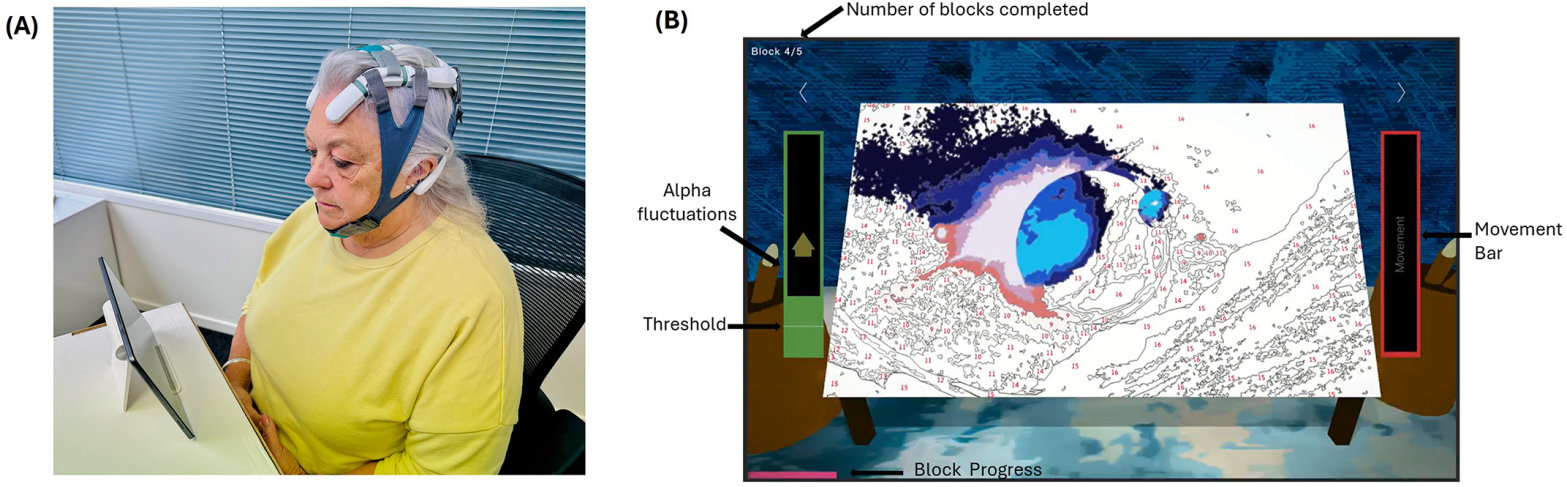
Home-based axon system. **(A)** Axon headset as worn by the user. **(B)** Screenshot of one of the games played by the user.

Participants were assigned a small, medium, or large Axon headset based on their head circumference to ensure electrode placement adhered to the 10–20 system (Figure 3A). Specifically, those with a head circumference of 520–539 mm received a small headset, those with 540–580 mm received a medium headset, and those with 581–620 mm received a large headset. NFB was delivered using a purpose-built, portable EEG headset (Axon system, Exsurgo Ltd, Auckland, New Zealand), linked via Bluetooth to a custom-built software application (Axon system, Exsurgo Ltd, Auckland, New Zealand) on a commercially available tablet (Samsung Galaxy, A7 SM-T505, Samsung, Suwon, South Korea). Participants were also provided with a tablet stand, charger, saline solution, chin strap and instruction manual.

### 2.3 Study outcomes

Outcome measurement data were collected at Week 0 (Initial/Pre-baseline assessments), Week 2 (Transition period), Week 11 (Post-intervention assessments) and Weeks 15, 19, and 23 (Follow-up assessments) (Figure 2). Primary outcome measures were reported changes in the Brief Pain Inventory (BPI) and Visual Analogue Pain Scale (VAS). The BPI measures pain severity and interference with daily activities. It includes items such as “Please rate your pain at its worst” on a scale from 0 (no pain) to 10 (pain as bad as you can imagine). Pain interference is assessed with items like “How much has pain interfered with your general activity?” scored on a similar 0–10 scale. The BPI has demonstrated excellent reliability (31). Secondary outcome measures were; (a) Depression, Anxiety, and Stress Scale (DASS21) comprising 21 items, divided into three subscales. Participants rated statements like “I found it hard to wind down” on a scale from 0 (did not apply to me at all) to 3 (applied to me very much or most of the time) (32); (b) Pain Catastrophizing Scale (PCS) measuring pain-related catastrophization via 13 items, including “I keep thinking about how much it hurts,” rated on a 5-point scale from 0 (not at all) to 4 (all the time), and comprising subscales for rumination, magnification, and helplessness, with a total score range of 0–52 (33); (c) Central Sensitization Index (CSI) measuring sensitization severity via 25 items, such as “I feel pain all over my body,” scored on a scale from 0 (never) to 4 (always), with a total score range from 0–100 (34); (d) Pittsburgh Sleep Quality Index (PSQI) measuring sleep dysfunction over the previous month via 7 components (e.g., sleep latency, sleep disturbances), with items such as “During the past month, how often have you had trouble sleeping because you wake up in the middle of the night or early morning?” and rated on a 4-point scale from 0 (not during the past month) to 3 (three or more times per week). The global score ranges from 0–21, with higher scores indicating poorer sleep quality (35), and; (e) Quality of Life (EQ-5D-5l) assessing health-related quality of life across five domains: mobility, self-care, usual activities, pain/discomfort, and anxiety/depression. Each domain has five levels, ranging from 1 (no problems) to 5 (extreme problems). It also includes a visual analog scale (EQ-VAS) where participants rate their overall health on a scale from 0 (worst imaginable health) to 100 (best imaginable health) (36). Changes in resting state relative alpha, theta, and high-beta activity from Week 1 to Week 22 were recorded and analyzed.

### 2.4 Neurofeedback training

After the initial assessments, participants received comprehensive training from the NFB clinician on all aspects of the study protocol, including headset fitting, orientation, application usage, equipment maintenance, training environment settings, and EEG artefact minimization. During this session, participants observed their real-time EEG activity. They were instructed to clench and unclench their jaws and blink their eyes to visualize the impact of these movements on the EEG recordings, thereby reinforcing the importance of minimizing movement during training. This also provided an opportunity to verify the correct functioning of the equipment through a graphical representation of their brain activity. Each participant was advised not to consume caffeine or sugary drinks for one hour beforehand, not to drink alcohol, and train when they felt most alert, depending on their daily routine. The training was considered complete once participants demonstrated proficiency in operating the equipment. Participants were instructed to conduct training sessions 5 times per week over an 8-week period (40 sessions). All training and technical support sessions were delivered via a secure video link. To monitor and improve compliance, email reminders were sent to participants when less than 5 sessions a week were performed, and technical support was provided when required. All data were collected remotely and stored securely in a cloud-based storage service (AWS) using end-to-end encryption. The trial was conducted entirely remotely.

### 2.5 Intervention

Each EEG baseline session commenced with a 2 min resting-state recording (eyes open) looking at a fixation cross on the screen, followed by a 2 min recording (eyes closed). During the intervention period, each session commenced with both baselines (as before) followed by the NFB training session. The participant's threshold for the game was calculated based on their eyes open EEG baseline recording at the beginning of each session, equating to 10% above their resting state relative alpha power. Each training session consisted of 5 × 5 min blocks with a one-minute rest period in between, with the option for a longer break if required. Participants viewed a gamified representation of their brain activity as the interface for self-regulation, choosing from five different “games” to play (Figure 3). The options for five different games are given below (Supplementary Data Sheet 1). The reward mechanism was identical for each of the games in that when the participant's relative alpha exceeded the baseline plus 10% threshold, the visual and audio feedback would occur via game progression:

• Jigsaw game—puzzle pieces are scattered randomly on the screen. As the participant exceeds the threshold, the pieces assemble into their correct positions one by one, completing the puzzle and a new puzzle appears.
• Balloon game—a balloon is situated on the ground. As the participant exceeds the threshold, the balloon ascends into the sky, revealing various background objects along the way.
• Lotus game—a lotus flower begins in a closed position. As the participant exceeds the threshold, the flower gradually opens, revealing all its petals. Multiple lotus flowers are available for the participant to cycle through.
• Bars game—three vertical bars represent relative theta, alpha, and high-beta power. When relative alpha increases above the 10% threshold, the alpha bar turns green; otherwise, it remains pink. As alpha increases *relative* to the other bands, theta and high beta also change.
• Paint game—the canvas starts as a painting by numbers. As the participant upregulates their relative alpha, the colors gradually appear on the canvas until the full picture is revealed, first as a painting and then as a full color picture, before randomizing into a new canvas.

At the end of the 5 blocks, there was a short break, followed by a 2 min baseline (eyes open) and the session ended, whereupon data was uploaded to the cloud for processing.

The audio and visual feedback was controlled by their real-time relative alpha fluctuations. Each time their relative alpha was 10% greater than their threshold, the game would progress. When the participants stayed over the threshold for 750 ms, they heard a single audio bell tone via the tablet's speaker. Thus, participants were rewarded visually for upregulating relative alpha and further rewarded with audio feedback for maintaining it, encouraging sustained neural firing within the target range located at C4, above the right hemisphere of the somatosensory cortex.

### 2.6 EEG processing

EEG signals were sampled from electrodes C4 and FP2, located above the somatosensory and prefrontal cortices, and transmitted to the Axon app via Bluetooth LE. Raw EEG signals from each electrode were captured at 250 Hz and bandpass filtered using multiple Infinity Impulse Response (IIR) filters. The frequency bands of interest were theta (4–8 Hz), alpha (8–13 Hz), beta (13–30 Hz) and high beta (20–30 Hz). The bands of interest and relative and absolute EEG band calculation were solely based on EEG activity at C4. The EEG activity at FP2 was only used for ocular artefact detection. Band-pass filtered data were examined for artefact signals. Artefacts were corrected online, using a custom-built algorithm, which subsequently discarded the contaminated portion. Artefacts were identified based on signal amplitude cutoffs specific to each frequency band. If the band-passed, filtered signal amplitude exceeded a predefined threshold, an amplitude correction was applied to mitigate the artefact. Additionally, the electrode contact quality was monitored in real-time using electrode impedance measurements. If impedance exceeded a set threshold for 4 s, the training was automatically paused, and the participant was prompted to adjust the headset fitment. During this process, real-time feedback on electrode contact quality was provided to guide the participant. These steps ensured that noisy EEG data were minimized and addressed promptly.

The bandpass filtered data for each frequency band of interest was squared and averaged over a 2 s window. The output of this step was the absolute power of each frequency band.

$$P_{\in }(F)=\;\frac{\sum_{i=1}^{n}\;x^{2}(i)}{n}$$

where: *i* = 1 to *n*; *x* = filtered EEG signal; *n* = window size of 500 samples (2 s of data at 250 samples/s); *P_ɛ_* (*F*) = absolute power of specific frequency (*F*).

The relative power of a particular EEG frequency band (*F*) was calculated by finding the ratio between absolute power and absolute broadband power of the concerned band, which was defined as the sum of the absolute powers of the theta, alpha and beta bands.

$$P_{\mathrm{rel}}(F)=\;\frac{P_{\in }(F)}{P_{\in }(\theta )+\;P_{\epsilon }(\alpha )+\;P_{\epsilon }(\beta )}$$

where *P*_rel_ (*F*) = relative power of specific frequency (*F*); *P*_ɛ_(*F*) = absolute power of specific frequency (*F*); *P*_ɛ_(*θ*) = absolute power of theta band; *P*_ɛ_(*α*) = absolute power of alpha band; *P*_ɛ_(*β*) = absolute power of beta band.

### 2.7 Data analysis

Data analysis was performed using SPSS (version 29.0, IBM, Chicago, IL). Primary and secondary outcome measures were analyzed in Week 0 (Pre-baseline), Week 2 (Pre-intervention), Week 11 (Post-intervention) and Week 15 (Follow-up). Data were presented as mean (±SE). Repeated measures ANOVA, followed by Bonferroni adjusted *post-hoc* analysis, were used to assess the effects of training sessions on different outcome measures. The improvement rate was calculated as the percentage change from pre-baseline:

$$\frac{\mathrm{Post}\;\mathrm{Intervention}-\mathrm{Pre}\;\mathrm{baseline}}{\mathrm{Pre}\;\mathrm{baseline}}\;\;\times 100$$

Clinically significant improvements were defined as ≥30% improvement from pre-baseline to post-intervention (37, 38). Last week's EEG data at post-intervention is compared with baseline EEG using a student's *t*-test. Linear least-squares regression was employed to estimate the regulation of relative alpha, theta, and high beta, with the Pearson correlation coefficient (*r*) used to determine the direction of EEG modulation. The significance level is set at *p* < 0.05.

## 3 Results

Eleven participants (6 female, 5 male) were recruited, one withdrew, and one was excluded due to insufficient sessions, leaving 9 participants' datasets in the final analysis. The entire cohort completed the pre-intervention baselines, the intervention period and the first follow-up period. On average, 9 participants completed 46 (±2.24) sessions over the 8-week intervention period (more than 40 sessions), corresponding to 98% adherence to the prescribed NFB training schedule. Three participants withdrew during the second and third follow-up periods, so only the first follow-up period (Week 11–15) was included for analysis. The mean age of participants was 51.89 (±6.85) years.

### 3.1 Primary outcomes

**Figure 4:**
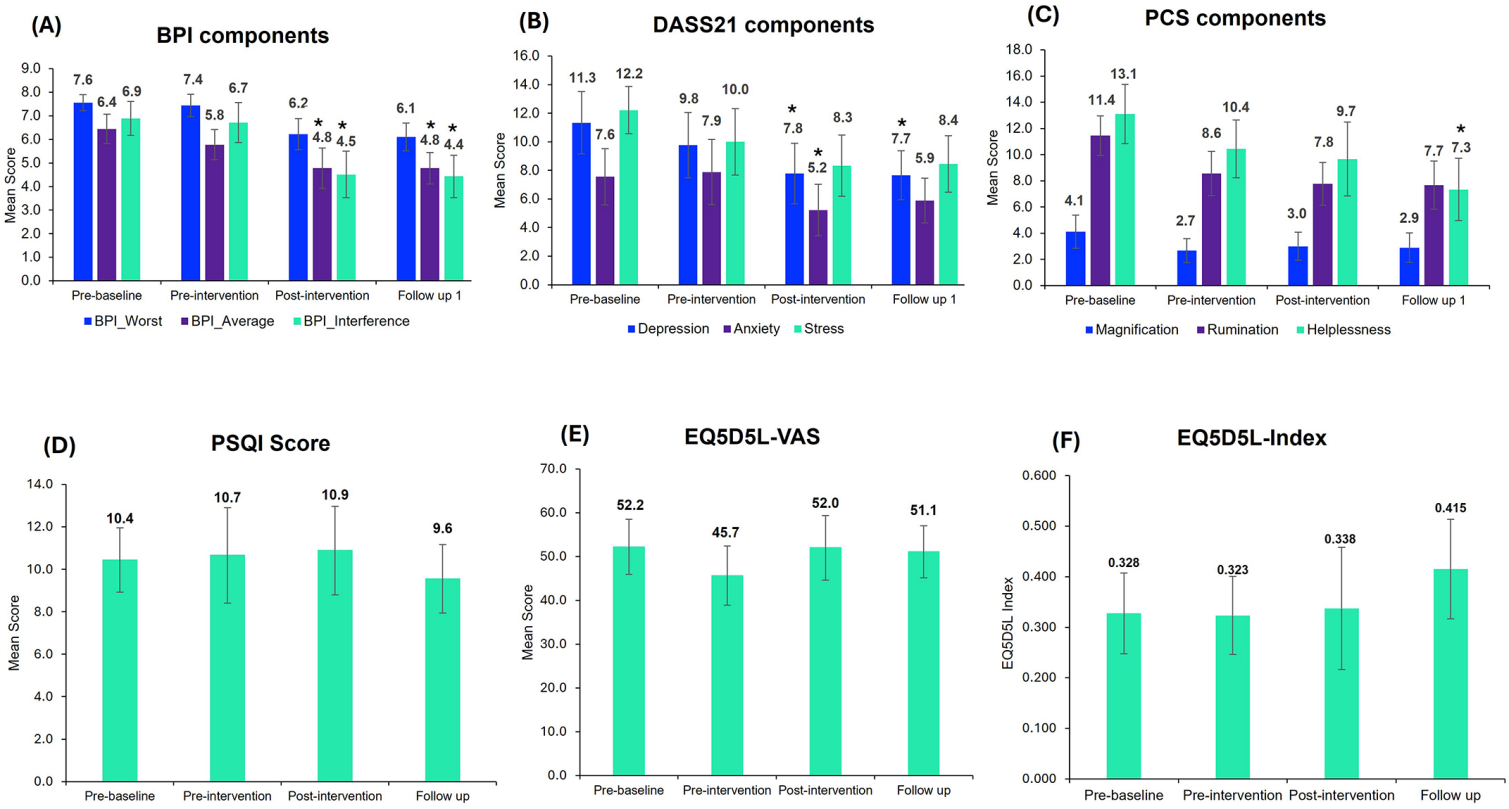
Outcome measurement scores in the trial. The above figure corresponds to the overall mean scores of the trial participants for **(A)** BPI, **(B)** DASS21, **(C)** PCS, **(D)** PSQI, **(E)** EQ-VAS and **(F)** EQ-5D-5l-Index. Bar graphs represent the mean ± standard error. Repeated measures ANOVA, followed by Bonferroni-adjusted *post-hoc* analysis, were used to identify significant changes compared to pre-baseline (*, *p* < 0.05). BPI, brief pain inventory; DASS21, depression, anxiety, stress; PCS, pain catastrophizing score; PSQI, Pittsburgh sleep quality index; EQ-5D-5l, quality of life questionnaires.

All 9 participants reported improvements in overall BPI (including BPI average, BPI worst and BPI interference) post-intervention. The results for primary outcomes are illustrated in Figure 4A. The four-level repeated measures ANOVA revealed a significant main effect for BPI average pain reduction over time, *F* (3, 24) = 5.65, *p* < 0.01, *η*_p_² = 0.41, BPI Worst, *F* (3, 24) = 5.37, *p* < 0.05, *η*_p_² = 0.28 and BPI interference, *F* (3, 24) = 8.25, *p* < 0.001, *η*_p_² = 0.51 indicating a large effect size. There were no significant main effects for the VAS score, *F* (3, 24) = 2.81, *p* = 0.06, *η*_p_² = 0.26. The *post-hoc* analysis confirmed that for the BPI average, significant pain reduction was observed between pre-baseline and post-intervention, *t* = 3.78, *p* < 0.05, Cohen's *d* = 0.79 and pre-baseline and follow-up, *t* = 3.54, *p* < 0.05, Cohen's *d* = 0.79. For BPI Interference, significant pain reduction was observed between pre-baseline and post-intervention, *t* = 3.92, *p* < 0.05, Cohen's *d* = 0.91 and pre-baseline and follow-up, *t* = 4.40, *p* < 0.05, Cohen's *d* = 0.94.

### 3.2 Secondary outcomes

Clinically significant (≥30%) improvements in BPI average pain were reported by 56% of participants (5 out of 9), 33% of participants (3 out of 9) had clinically significant improvements in the worst pain, and 44% of participants (4 out of 9) experienced clinically significant improvements in overall pain scores.

The four-level repeated measures ANOVA was conducted to assess changes in DASS21 components including depression, anxiety and stress across the four-time points (pre-baseline, pre-intervention, post-intervention and follow-up). The analysis revealed a significant effect of time on DASS21, *F* (3, 24) = 5.16, *p* < 0.05, *η*_p_² = 0.39, Depression, *F* (3, 24) = 3.60, *p* < 0.05, *η*_p_² = 0.31 and Stress, *F* (3, 24) = 4.58, *p* < 0.05, *η*_p_² = 0.36. There were no significant effects for Anxiety, *F* (3, 24) = 2.68, *p* = 0.07, *η*_p_² = 0.25. The comparison between time points for DASS21 components is illustrated in Figure 4B.

The four-level repeated measures ANOVA revealed a significant main effect for PCS, *F* (3, 24) = 5.62, *p* < 0.01, *η*_p_² = 0.41, Rumination, *F* (3, 24) = 5.39, *p* < 0.01, *η*_p_² = 0.40 and Helplessness, *F* (3, 24) = 6.51, *p* < 0.01, *η*_p_² = 0.45. There were no significant effects for the Magnification, *F* (3, 24) = 1.217, *p* = 0.32, *η*_p_² = 0.13. The changes in PCS component scores at different time points are represented in Figure 4C.

**Figure 5:**
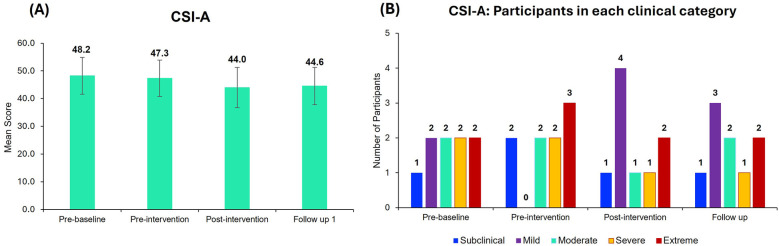
Central sensitization inventory part A (CSI-A) scores. **(A)** Mean CSI-A scores for all the 9 participants at different time points. Bar graphs represent the mean ± standard error. **(B)** Number of participants in each category (Subclinical, mild, moderate, severe, and extreme) of the CSI-A across different time points. Most of the participants are categorized under the mild clinical category after neurofeedback treatment.

Although the mean CSI scores improved from pre-baseline to post-intervention and at follow-up, the improvements were not statistically significant (Figure 5A). At the pre-baseline, participants exhibited mild to extreme levels of central sensitization (CSI A score > 33) whereas after the intervention, participants were mostly categorized under the mild category at the post-intervention and follow-up points (Figure 5B).

No change in the sleep score was observed (Figure 4D). Although there was no change in the overall mean PSQI score, 4 out of 9 participants improved their sleep score, with 33% showing ≥30% significant improvements.

EQ-5D-5L scores were similar at different time points (Figures 4E, F), with no significant differences observed.

### 3.3 Modulation in EEG bands

**Figure 6:**
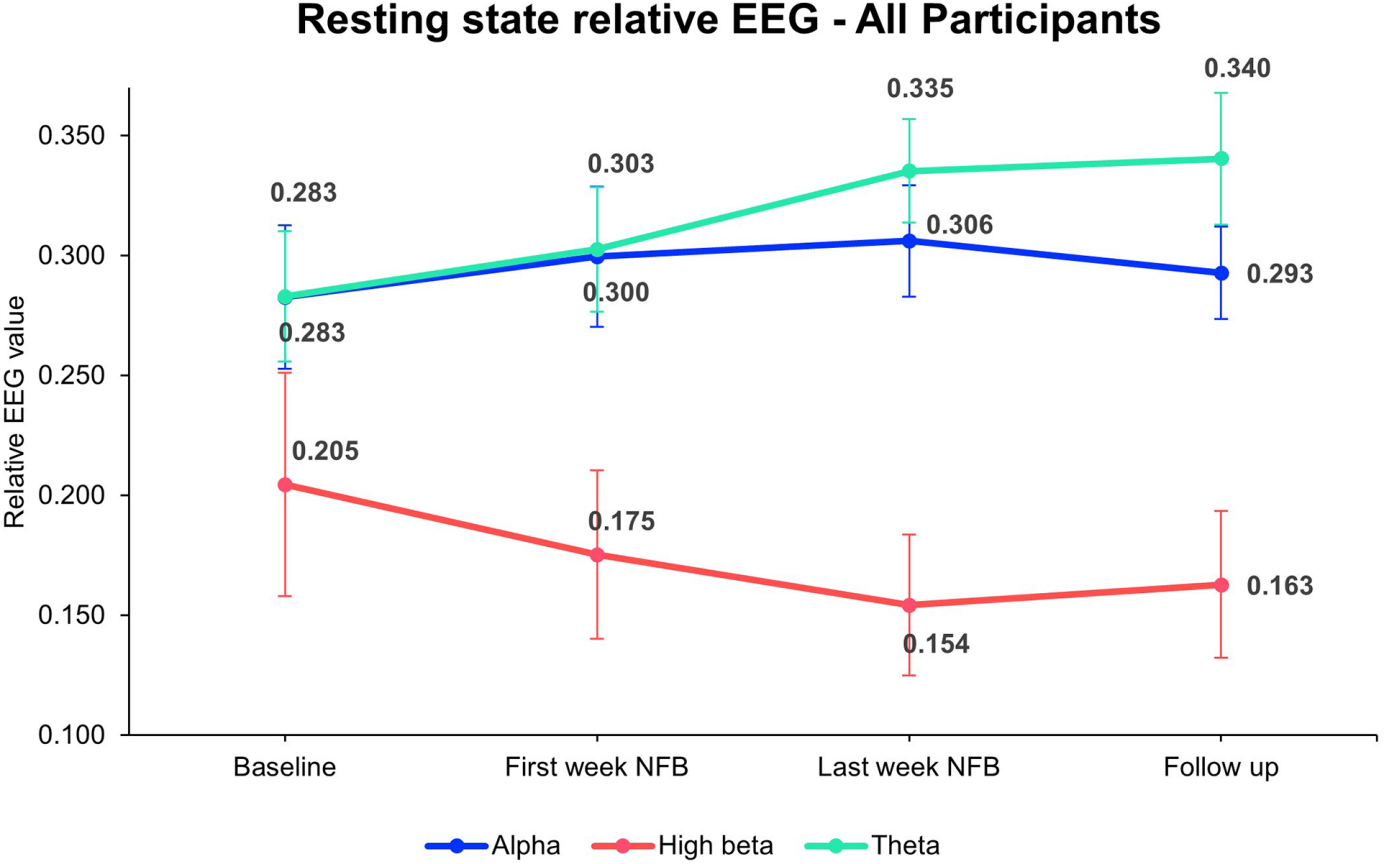
Overall mean resting-state relative EEG bands. The figure represents resting-state relative EEG power across all participants measured at four time points: Baseline, first week of NFB, last week of NFB, and after follow-up week. The values for Alpha (blue line), High Beta (red line), and Theta (green line) bands are shown with mean values and error bars representing SE. The data indicates changes in brainwave activity over the course of the intervention, with notable trends observed in each frequency band. For statistical analysis EEG data at last week NFB is compared with the baseline All 9 participants' average data were included from baseline till last week NFB. NFB, neurofeedback; SE, standard error.

Modulation of resting-state relative alpha, high beta, and theta were observed. The group mean of EEG bands at different time points is depicted in Figure 6. A paired *t*-test between baseline and post-intervention indicated an increase in relative alpha (*t* = −1.96, *p* = 0.08, Cohen's *d* = −0.65) and theta (*t* = −2.05, *p* = 0.07, Cohen's *d* = −0.68) and a decrease in relative high-beta post-intervention (*t* = 2.08, *p* = 0.07, Cohen's *d* = 0.69), however, the changes were not statistically significant.

**Figure 7:**
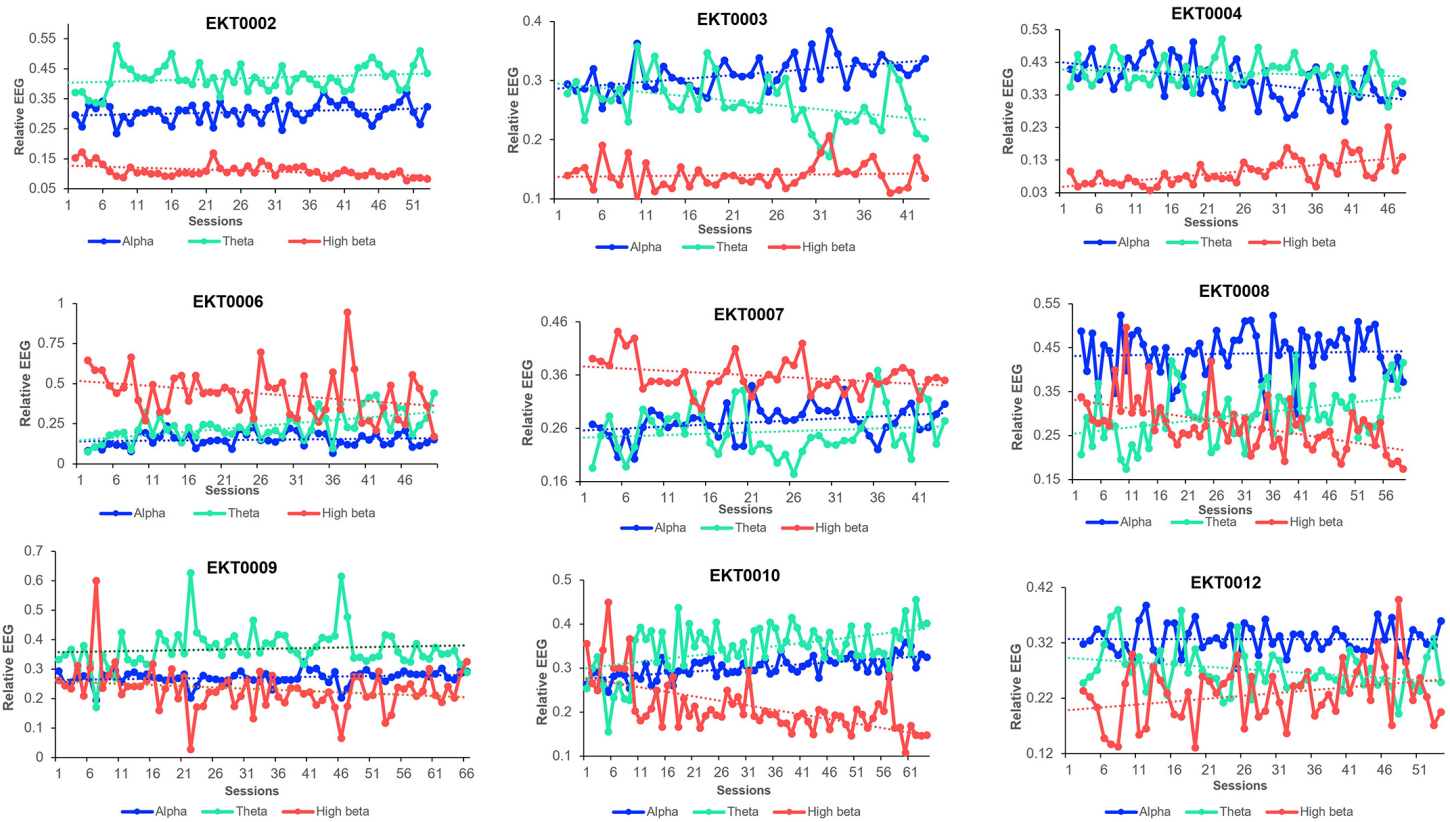
Resting-state relative EEG bands. Trends in resting-state relative alpha, theta, and high beta bands in each participant throughout the trial study.

The individual per-session resting-state relative EEG for each participant is displayed in Figure 7, with upward/downward EEG trends depicted in Table 2. Of the 9 participants included in the analysis, 7 showed an improved BPI average with 5 exhibiting increased relative alpha activity from pre-baseline to post-intervention. Of these, 4 participants demonstrated a significant upward trend between sessions performed and relative alpha (*p* < 0.05), indicating most participants who upregulated relative alpha experienced reductions in pain. Six participants who decreased their resting-state relative high beta improved their depression scores, with 5 of them showing a significant downward trend (*p* < 0.05). Similarly, out of the 6 participants who improved their anxiety scores, 4 showed a significant downward trend of relative high beta. Additionally, among the 7 participants who improved their DASS21 scores, 5 significantly downregulated relative high beta, indicating participants who downregulated relative high beta experienced an improvement in mood.

**Table 2:** EEG trends in each participant during 8 weeks of neurofeedback training.

Participant	Relative EEG Bands	Pearson's R	*p*-value	EEG Trends
KT0002	Alpha	0.227	0.105	UP
	High beta	−0.480	*<0*.*001*	***DOWN*** ***
	Theta	0.206	0.142	UP
EKT0003	Alpha	0.494	*<0*.*001*	***UP*** ***
	High beta	0.074	0.643	No change
	Theta	−0.418	*0*.*006*	***DOWN*** **
EKT0004	Alpha	−0.530	*<0*.*001*	***DOWN*** ***
	High beta	0.635	*<0*.*001*	***UP*** ***
	Theta	−0.143	0.336	No change
EKT0006	Alpha	0.138	0.958	No change
	High beta	−0.306	*0*.*032*	***DOWN*** *
	Theta	0.575	*<0*.*001*	*UP*
EKT0007	Alpha	0.320	*0*.*036*	***UP*** *
	High beta	−0.325	*0*.*034*	***DOWN*** *
	Theta	0.139	0.375	No change
EKT0008	Alpha	0.059	0.661	No change
	High beta	−0.552	*<0*.*001*	***DOWN*** ***
	Theta	0.394	*0*.*002*	***UP*** *
EKT0009	Alpha	0.296	*0*.*015*	***UP*** *
	High beta	−0.131	0.291	No change
	Theta	0.042	0.737	No change
EKT0010	Alpha	0.666	*<0*.*001*	***UP*** ***
	High beta	−0.647	*<0*.*001*	***DOWN*** ***
	Theta	0.511	*<0*.*001*	*UP*
EKT0012	Alpha	−0.002	0.989	No change
	High beta	0.295	*0*.*034*	***UP*** *
	Theta	−0.304	*0*.*028*	***DOWN*** *

Asterisks represent significant upward or downward trends.

* *p* < 0.05.

** *p* < 0.01.

*** *p* < 0.001.

### 3.4 Medication

Medication was not altered during the trial. All participants were on pain medication, and many were also taking a range of medications including antidepressants, anticonvulsants, anti-anxiety medications, opioids, and sleep medications. For further information, refer to the feasibility and safety publication of this trial for a comprehensive list (30).

## 4 Discussion

The primary objective of this study was to examine the effectiveness of an 8-week home-based NFB intervention for individuals living with CNP within the NHS treatment framework. The study aimed to evaluate whether the intervention could achieve a reduction in pain as the primary outcome and improvements in secondary outcomes, including mood, sleep, central sensitization, and quality of life.

This prospective interventional study included nine participants who completed the intervention, demonstrating significant improvements in pain and psychological well-being. Seven (78%) reported improvements in BPI average pain scores and VAS pain scores. Results aligned with the first hypothesis, showing reductions in self-reported pain levels and improvements in mood in terms of depression, anxiety and PCS. These findings suggest that NFB, in conjunction with usual care, offers promising adjunct treatment for individuals with treatment-resistant CNP and comorbidities. Regarding the second hypothesis, changes in resting-state EEG activity were observed over the course of the intervention. The EEG bands of interest showed changes in the frequencies known to be associated with chronic pain and associated symptomatology (18–20, 22, 23), providing valuable objective evidence alongside subjective improvements. The third hypothesis was supported by the data, demonstrating an association between resting-state relative alpha and pain, as well as resting-state relative high beta and mood scores. These results indicate that the observed neuroplastic changes in these EEG bands may contribute to alleviating the pain, as well as the emotional and cognitive aspects of CNP.

A notable gender-specific variation was observed, as most of the trial participants were female with a mean age of 51.17 (±9.66) years compared to males with a mean age of 53.33 (±13.13) years. This gender difference reflects previous research suggesting women tend to suffer from chronic pain more frequently (39, 40), and that CNP is more frequent in women (8% vs. 5% in men) and in middle-aged people (>50 years of age) (41, 42).

The main findings indicate that the NFB intervention had a meaningful impact on pain outcomes for this pilot cohort. All participants reported significant improvements in BPI along with pain interference, demonstrating the efficacy of the intervention in addressing the overall pain experience and its interference with daily activities, consistent with a previous proof of concept study using the same NFB system and protocol (29). Clinically significant improvements in BPI average was observed in 5 participants (out of 9). Furthermore, the improvement in VAS scores aligned with the positive trends observed in BPI scores, indicating harmonization across outcome measures with respect to the impact on perceived pain intensity.

The secondary outcomes showed notable improvements in depression, anxiety, stress, and pain catastrophizing. The significant impact on anxiety, depression and stress scores, post-intervention and at follow-up, would benefit from further investigation with a larger cohort, and the improvements in CSI scores would benefit from further investigation as a contribution to overall psychological well-being. While no significant change in sleep and quality of life measures were observed, the examination of individual participants' data revealed variations in scores, which suggests that a larger sample size might yield different results. The observed improvement in secondary outcomes, such as depression, may be attributed to the interconnected nature of neurophysiological networks involved in pain and mood regulation. Chronic pain and depression share overlapping neural circuits, particularly in the anterior cingulate cortex, prefrontal cortex, and insula. NFB training targeting alpha activity may enhance self-regulation within these networks, indirectly improving mood symptoms. Furthermore, the relaxation and increased attentional control associated with alpha modulation could contribute to a reduction in depressive symptoms, even if these were not the primary targets of the intervention. However, we acknowledge that the absence of a control group limits our ability to exclude placebo effects as a potential contributor to these improvements.

The modulation of the targeted EEG bands showed encouraging changes which require further investigation. The increase in resting-state relative alpha activity aligned with previous studies, where higher alpha power was associated with relaxation and reduced pain perception (29, 43). The concurrent improvements in pain and psychological outcomes among participants who exhibited increased relative alpha further support the role of an alpha modulation NFB protocol for CNP. The reduction in high beta activity is also noteworthy, considering its association with heightened arousal and stress (44). The observed association between decreased high beta and improvement in psychological distress scores highlights the potential relevance of high beta modulation in enhancing mental well-being. Changes in resting-state EEG activity serve as markers of structural and functional brain modulation, indicating that neuroplastic adaptations occurred due to the intervention. CP can progressively alter brain representations, redirecting sensory pathways toward emotional networks and the limbic system (45, 46). The interconnectedness of the networks associated with CP, often referred to as the “pain matrix,” suggests that the modulation of one neural region can trigger downstream effects across adjacent areas (47, 48).

Importantly, some patients experience a heavier emotional weighting to their pain, which may be more closely associated with beta band activity than with alpha. However, the modulation of one frequency band relative to others typically leads to adjustments in other bands (49). For instance, the observed upregulation of relative alpha in our cohort appears to have modulated beta and/or theta bands between sessions, highlighting the dynamic interplay between these networks. It is known that theta and beta have an inverse relationship affecting EEG activity during neuromodulation (50). In our study, we have observed that when relative alpha is upregulated, relative theta and beta (especially high beta) most commonly move in relation to each other. Specifically, if theta is upregulated, high beta is invariably downregulated. While this interaction can also occur between alpha and theta or alpha and high beta, the inverse relationship between theta and high beta is the most pronounced. These changes likely influenced whole-brain networks linked to the primary and secondary symptoms of CP. Specifically, the modulation of the somatosensory cortex at C4 likely suppressed neuronal hyperexcitability, realigning the imbalance between ascending nociceptive input and descending inhibitory control within this region (51). Recent studies suggest that such modulation leads to neuroplastic changes (52), as reflected in the altered resting-state activity observed in this study. Future investigations should further explore this dynamic by analyzing changes across frequency bands and incorporating longitudinal structural and functional neuroimaging techniques (e.g., fMRI, MEG) to elucidate these mechanisms.

Interestingly, resting-state relative theta was quite high among all the participants and exhibited an increasing upward trend throughout the trial period. Increased resting state theta activity has been reported in neuropathic pain patients (24, 53). The neural source of increased theta power is suggested to be located in the parts of the “pain matrix” such as the prefrontal medial and anterior cingulate cortex (54). Additionally, studies have suggested that internally generated abnormal firing of neurons may disrupt thalamocortical networks and lead to abnormal pain processing, especially in neuropathic pain conditions, where a degree of thalamic denervation has been observed (55–57). Reduced thalamic inhibition appears to be associated with increased neural activity at around 4 Hz, serving as the origin of the elevated theta power. This occurrence is referred to as Thalamocortical Dysrhythmia (24, 58). The high theta activity observed in our trial participants suggests that increased theta power may represent a biomarker of CNP.

The emergence of theta activity from thalamic neurons has revealed two distinct components of pain perception in individuals with central pain syndromes. One aspect involves the physical sensation of pain in specific regions, notably the somatosensory cortex. The other aspect concerns the emotional aspect of pain perception, involving complex neural networks akin to thalamocortical “loops”. This emotional aspect of pain, often characterized as a “moral pain” associated with feelings of being wounded, is consistently observed in chronic pain patients (53). Thus, high theta activity has been implicated in emotional dysregulation and may affect the quality of life in CP patients. In this study, we observed that participants did not exhibit improvement in their overall quality of life scores, suggesting that 8 weeks of the intervention period was not long enough to elicit transferability from symptom improvement to quality of life measures in this cohort with complex symptoms.

The individualized analysis of EEG trends revealed that participants who upregulated relative alpha or downregulated relative high beta demonstrated concurrent improvements in pain and psychological distress. Although the modulation of EEG bands did not reach statistical significance at the group level, the improvements observed at the individual level underscore the importance of focusing on personalized outcomes in pain interventions. This aligns with a broader paradigm shift in the field, where clinical relevance and individual variability are increasingly prioritized over sole reliance on group-based statistical measures. Such an approach is particularly pertinent in CP management, where patient-specific factors often dictate treatment efficacy.

## 5 Limitations

The obvious limitation of this study is the small sample size, which makes it difficult to infer clinically meaningful changes. However, the primary objective was to evaluate the feasibility and initial efficacy of the NFB protocol in a home-based setting, which required a smaller cohort for manageable monitoring. Despite the limited sample size, the results have been sufficiently positive to warrant a further randomized controlled trial with a larger cohort of CNP patients. Another limitation is that participants were using various pain medications, and some were also taking anticonvulsants and antidepressants. Studies have reported that pain medications commonly prescribed for chronic pain affect EEG activity (59) and this would also have influenced the data. It is acknowledged that medication is crucial in the management of CNP, so gaining further knowledge as to how different prescribed medications influence EEG activity would be advantageous when considering treatment effects and intervention periods. It would also be useful to consider lengthening the duration of the NFB intervention period, which might yield improved outcomes for individual patients.

## 6 Conclusion

In conclusion, this trial study provides preliminary evidence supporting the efficacy of NFB in alleviating the symptoms of CNP and demonstrates significant improvements in various psychological domains, offering a promising approach to addressing the complex interplay between CNP and psychological distress in treatment-resistant patients. The association between EEG changes and clinical outcomes suggests a promising avenue for future research in a larger cohort.

## Data Availability

The original contributions presented in the study are included in the article/Supplementary Material, further inquiries can be directed to the corresponding author.
