# Supplementary material for: A home-based EEG neurofeedback treatment for chronic neuropathic pain—a pilot study
Source: Front Pain Res (Lausanne). 2025 Mar 11;6:1479914. doi: 10.3389/fpain.2025.1479914 (PMC11933074; doi:10.3389/fpain.2025.1479914)
Supplement: Supplementary file 1 [file Datasheet1.docx]

Supplementary Material

# Supplementary Figures


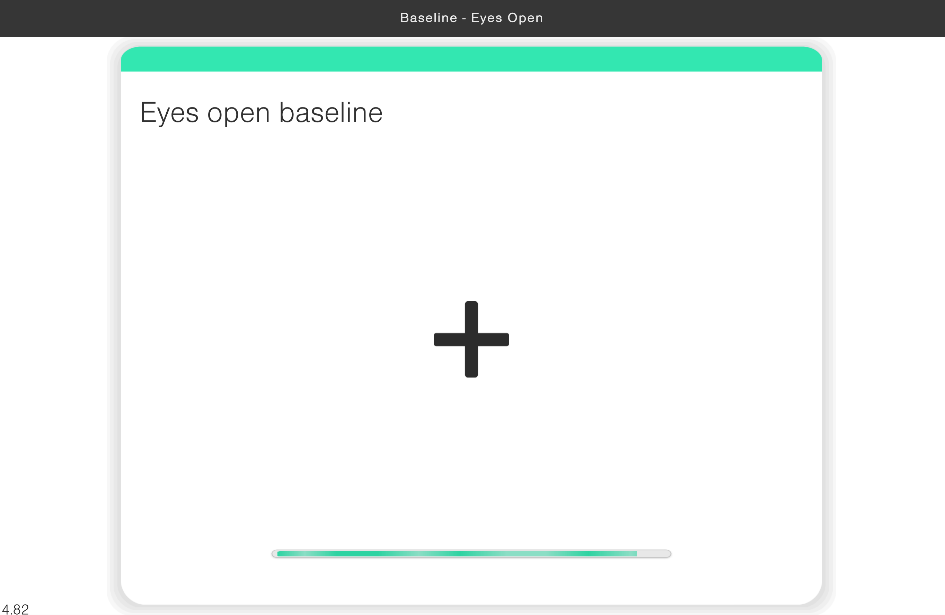


**Supplementary Figure 1.** Screenshot of Baseline eyes open screen in the Axon app.


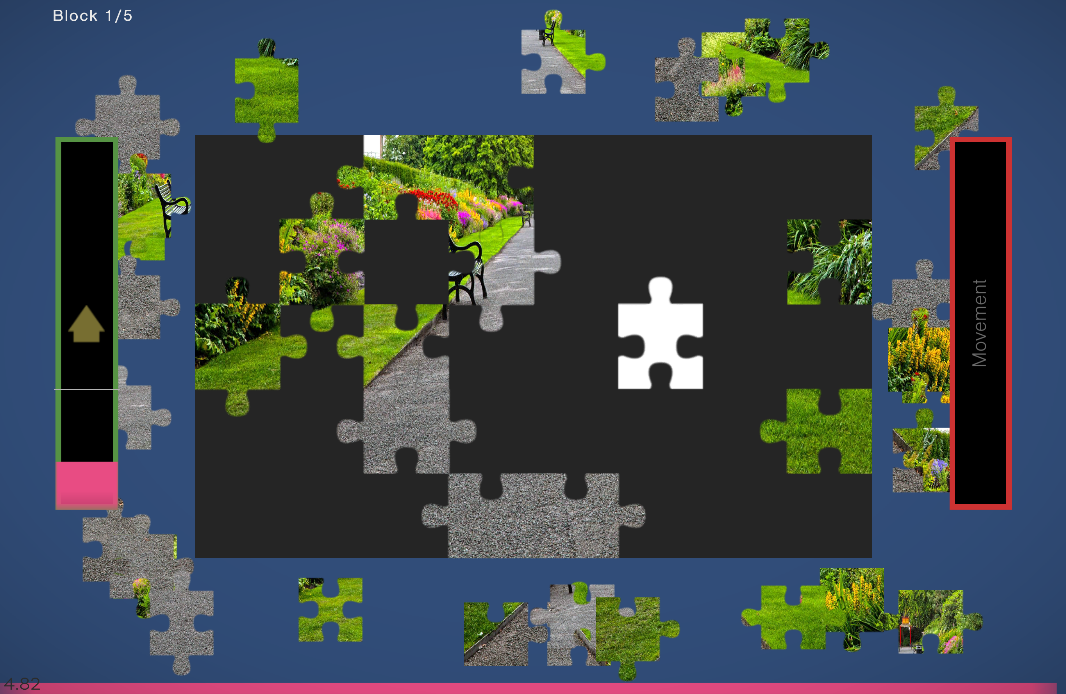


**Supplementary Figure 2.** The game screen of Puzzle game.


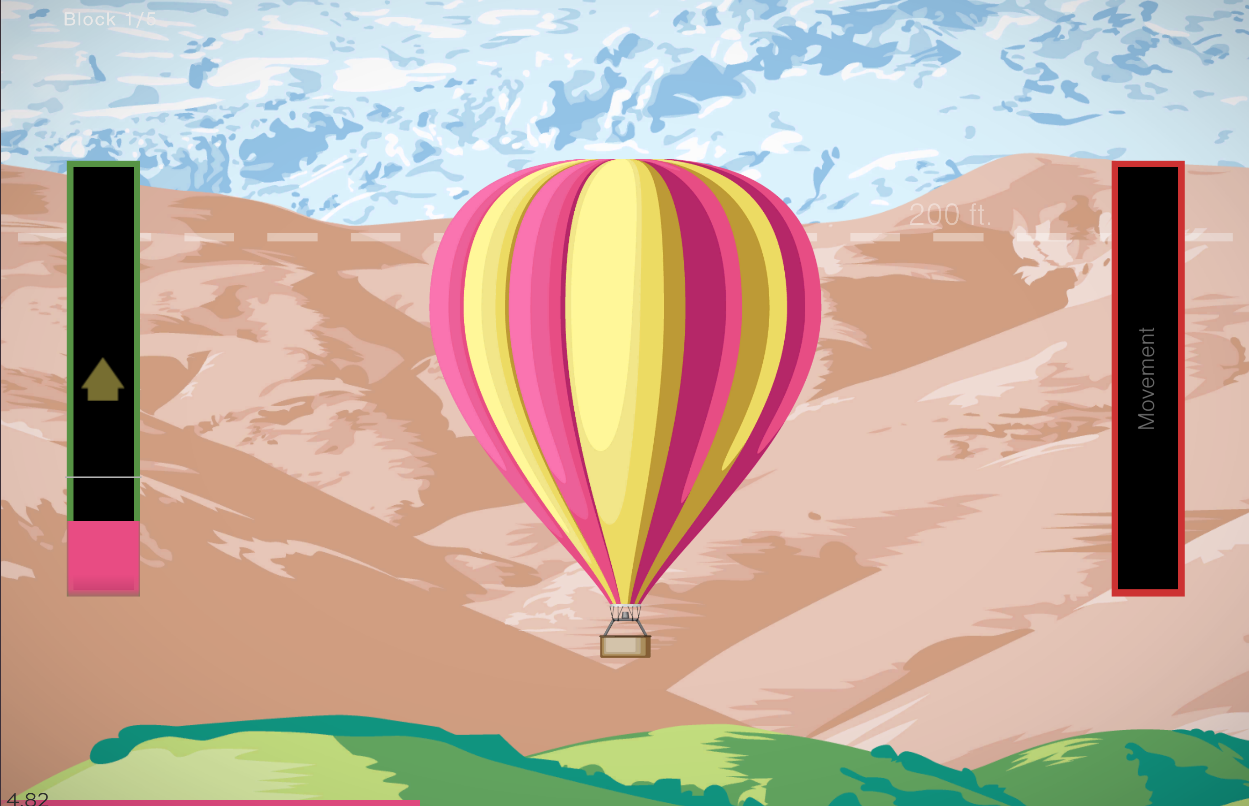


**Supplementary Figure 3.** The game screen of Ballon game


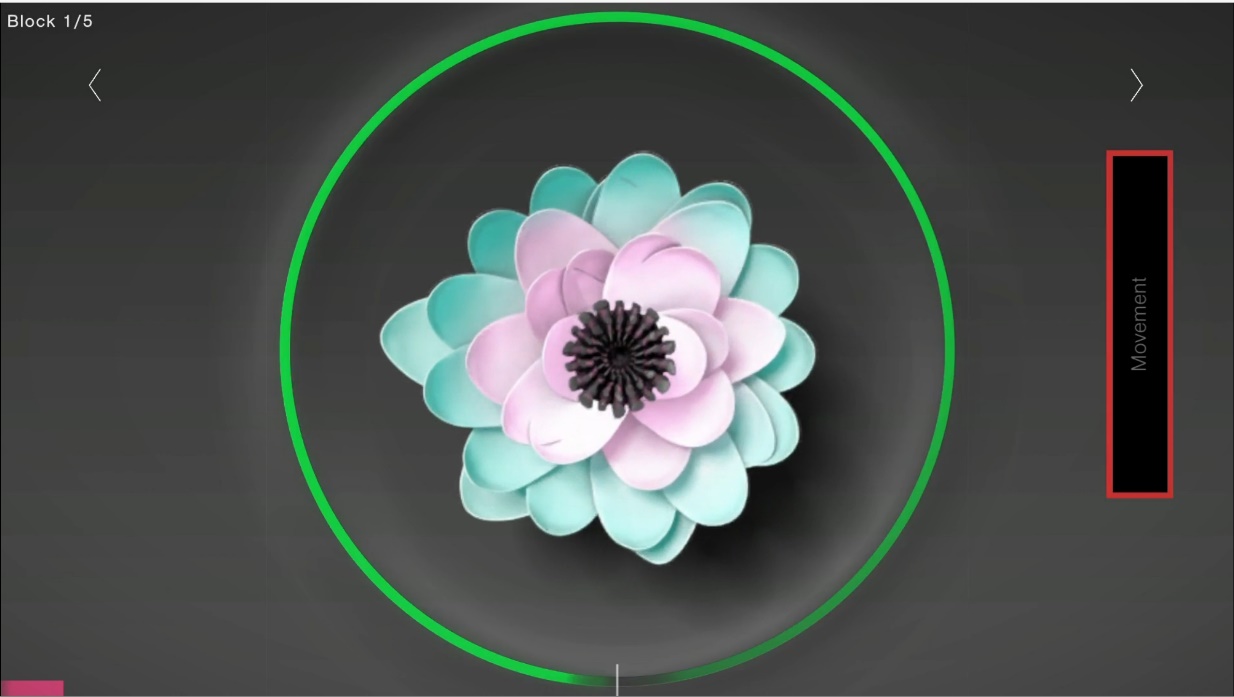


**Supplementary Figure 4.** The game screen of Lotus game.


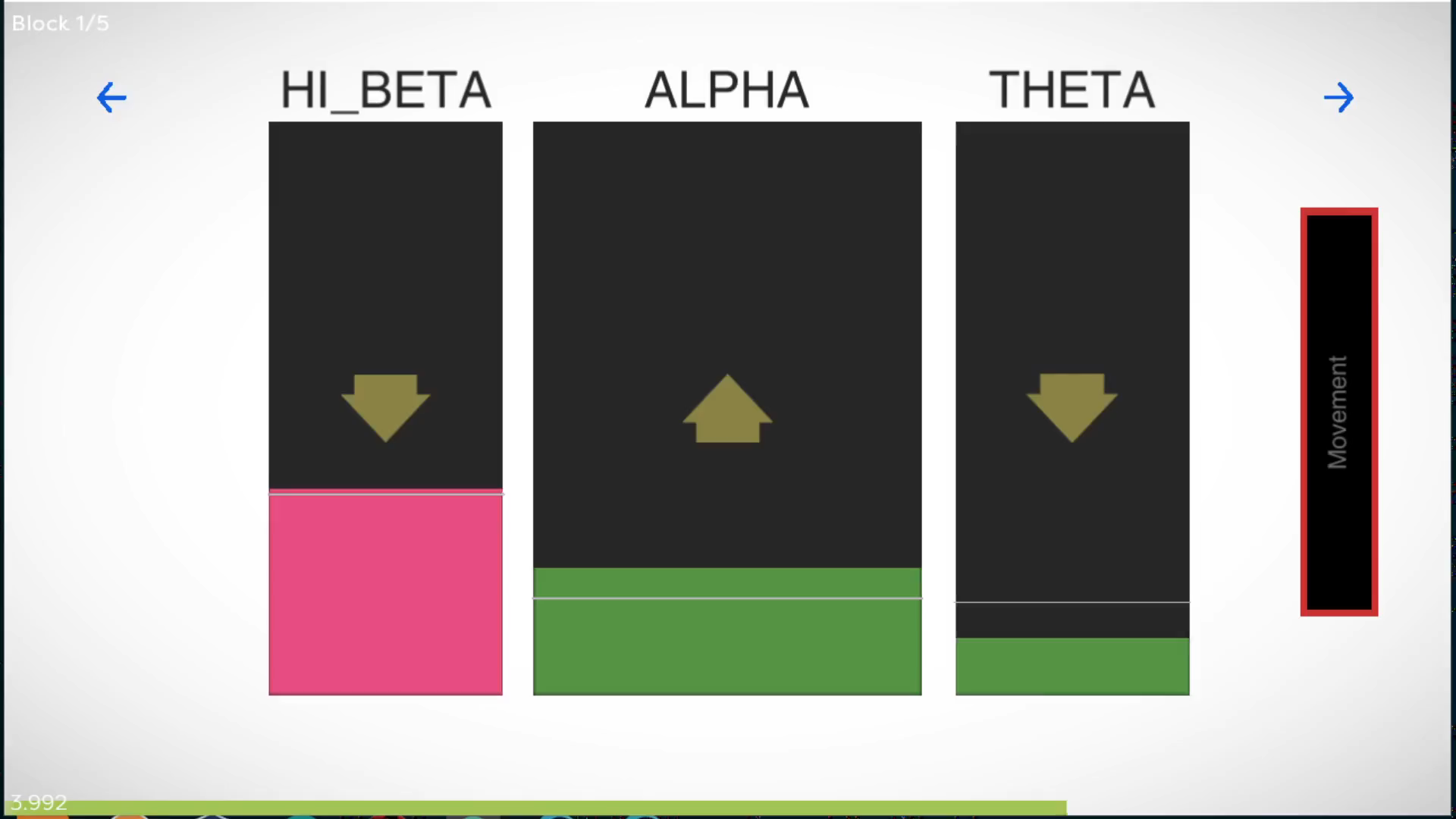


**Supplementary Figure 5.** The game screen of Bars game


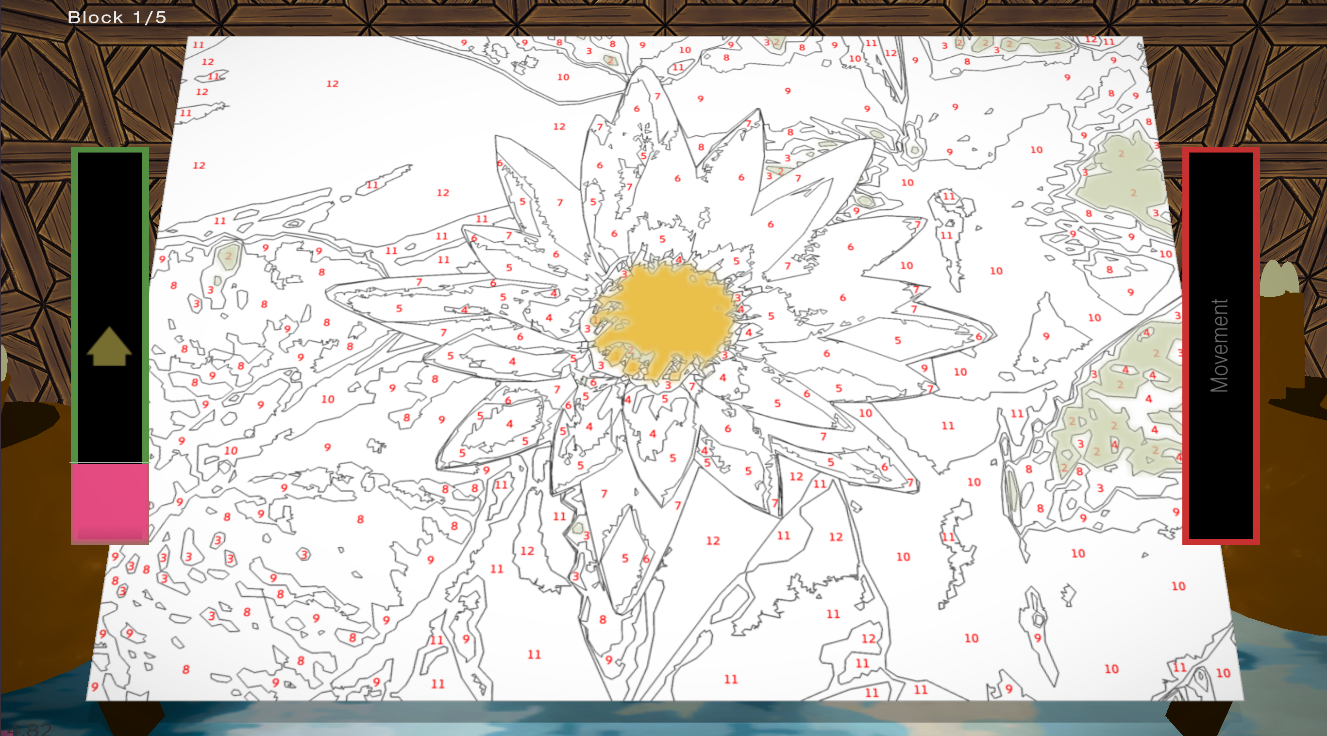


**Supplementary Figure 6.** The game screen of Paint game.
